# Community health workers fostering trust and engagement in a community-based intervention for depressive older adults in Peru: a qualitative study

**DOI:** 10.21203/rs.3.rs-7665993/v1

**Published:** 2025-10-19

**Authors:** Diego Otero-Oyague, Dafne Lastra, Vanessa Patiño, Ivonne Carrión, Tatiana Cruz-Riquelme, Suzanne L. Pollard, José F. Parodi, Lesley Steinman, Joseph J. Gallo, Rubén Valle, Nicolas Castro, Oscar Flores-Flores

**Affiliations:** Universidad Antonio Ruiz de Montoya, Escuela de Psicología; Universidad de San Martin de Porres; Asociación Benéfica PRISMA; Universidad de San Martin de Porres; Universidad de San Martin de Porres; Johns Hopkins University; Universidad de San Martin de Porres; University of Washington School of Public Health; Johns Hopkins Bloomberg School of Public Health; Universidad de San Martin de Porres; Universidad Antonio Ruiz de Montoya, Escuela de Psicología; Universidad de San Martin de Porres

**Keywords:** Older Adults, Community Health Worker, Community-based intervention, Acceptability, Feasibility, Trust

## Abstract

**Background:**

Innovative community-driven approaches, such as task-sharing interventions with Community Health Workers (CHWs), are essential to reduce the mental health care gap. This study explored how CHWs’ actions, experiences, knowledge, and resources contributed to fostering the acceptability and feasibility of a community-based mental health intervention (VIDACTIVA) for older adults.

**Methods:**

We examined the role of CHWs (n = 16) delivering VIDACTIVA, an evidence-based CHW-led intervention for older adults with depression. VIDACTIVA integrates Problem-solving therapy and Behavioral Activation in eight home-based sessions. Data included in-depth interviews and field notes collected across a 3-year period of piloting, adaptation, and implementation. An inductive thematic analysis identified key CHW practices that enhanced the intervention’s engagement.

**Results:**

Sixteen CHWs (aged 39–72, median 60 years) participated. Three main themes emerged as central: (1) establishing trust with older adults through active listening, empathy, and addressing urgent needs; (2) adapting session settings and creatively introducing tools to sustain engagement; and (3) fostering strong peer support networks that enhanced CHWs’ confidence, learning, and commitment throughout implementation.

**Conclusion:**

CHWs play a pivotal role in community-based mental health interventions by building trust with participants, leveraging their community knowledge, and working as a collective that supports learning, self-care, and teamwork, factors that humanize care and strengthen the sustainability of programs.

**Trial registration:**

The current trial registration number is NCT06065020, which was registered on 26th September

## Background

Late-life depression is one of the most common mental health conditions among older adults and represents a major public health concern worldwide. A recent systematic review and meta-analysis estimated that nearly one in three older adults experience depressive symptoms globally (1). In Peru, the prevalence of depression among older adults is estimated between 10% and 15% according to the 2020 Demographic and Family Health Survey (2). Marked inequities have also been documented: prevalence reaches 30% among older adults with low income compared to 15% among those with middle or higher income (3). In urban resource-constrained settings, broader social determinants such as poverty, unsafe housing, limited health infrastructure, mental health stigma and ageism further exacerbate the risk of depression (4–6). These challenges are worsened by the scarcity of mental health professionals and trained providers, leaving many older adults without timely or appropriate care.

To address this gap, the World Health Organization has promoted task-shifting and task-sharing interventions as strategies to integrate non-specialists into routine health care and expand access in low- and middle-income countries (7). Community Health Workers (CHWs) have been increasingly recognized as trusted contributors to mental health interventions, with evidence supporting their roles in prevention, treatment, and ongoing support across diverse contexts (8, 9).

Trusted and embedded within communities, CHWs bridge gaps between health systems and populations, and their involvement in the design and delivery of community-based programs enhances acceptability, feasibility, sustainability, and effectiveness (10–18).

In Peru, CHWs, known locally as *agentes comunitarios de salud*, are voluntary individuals recognized by their communities and formally registered by health authorities. Traditionally, CHW extend access to healthcare services by serving as bridges between community members, formal health professionals, and institutions, contributing to areas such as infection disease prevention, and maternal and child health (19, 20). However, it has been less common for CHWs to lead the delivery of interventions themselves, particularly in the field of mental health (14, 21).

A pilot trial of VIDACTIVA, a multi-component community-based intervention delivered by CHWs that included eight sessions of a low-intensity psychological program for older adults with depressive symptoms, has been conducted (22). As part of its design, the program incorporated core components from the *Program to Encourage Active, Rewarding Lives* (PEARLS), an evidence-based intervention originally developed at the University of Washington. Specifically, VIDACTIVA integrated PEARLS’ focus on problem-solving skills and behavioral activation, two approaches shown to be effective and feasible in home-based interventions for older adults with depression in the United States (23), and combined them with locally relevant strategies and the active role of CHWs to respond to the Peruvian context. This study examined the role and contributions of CHWs in fostering trust and engagement in the intervention. Specifically, we analyze the actions, experiences, knowledge, and resources mobilized by CHWs as strategies to strengthen their bonds with older adult participants and to facilitate the delivery of VIDACTIVA. We also reflect on the practical implications of these strategies for the design and implementation of community-based interventions in similar settings.

## Methods

### Study Setting

This study was conducted in Villa El Salvador (VES), an urban low-income district located in the south of Lima, Peru, mostly populated by long-standing migrants from the Andean Peruvian central and south regions that arrived between 1970 and 1980 due to insecurity, poverty and seeking better socio-economic opportunities. In Peru, formal mental health services are delivered through primary healthcare centers (PHCs) and community mental health centers (CMHCs). PHCs typically manage mild cases and may or may not have a mental health professional on staff (24). More moderate to severe cases are referred to CMHCs, which have multidisciplinary teams that include psychiatrists and other mental health specialists. In 2024, there were 3 CMHCs and 16 primary health centers in VES, many of which were supported by a network of CHWs (22).

### Study Design

This qualitative study drew on multiple sources of data, including in-depth interviews and field notes, to analyze CHWs’ experiences and perspectives over a three-year period of piloting, adaptation, and implementation of the VIDACTIVA intervention.

### The VIDACTIVA intervention

**Figure 1:**
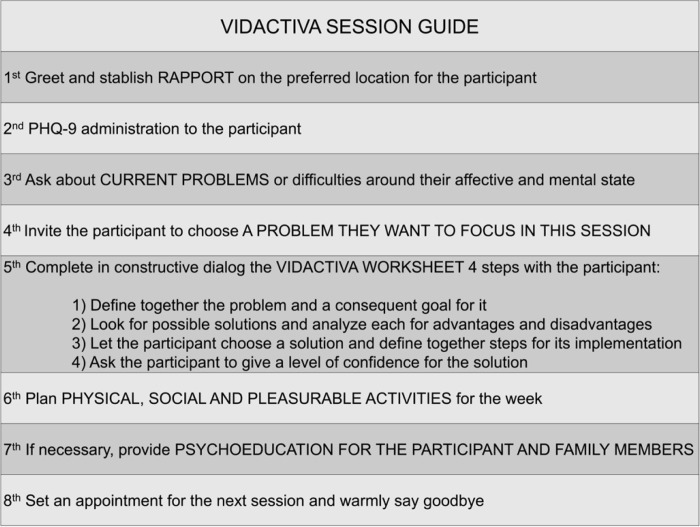
English version for the VIDACTIVA session guide used by CHWs on field

In summary, VIDACTIVA consists of eight in-person sessions delivered by pairs CHWs over a 14-week period (reference anonymized). The sessions are semi-structured and conducted either in the participant’s home or in a convenient community location, such as a relative’s home or a public park (Fig. 1). In addition, weekly supervision meetings were held with the group of CHWs, facilitated by a clinical psychologist and a general practitioner. During these meetings, CHWs shared their experiences with participants, discussed challenges, uncertainties, and initiatives that emerged in relation to each VIDACTIVA session, and engaged in reflective and collaborative dialogue. These spaces allowed CHWs to explore the meaning of their work and to collectively identify lessons learned throughout the intervention.

### Participants

A total of 31 CHWs were initially recruited through open calls disseminated by the local health network and community channels to participate in the adaptation and design of VIDACTIVA. All attended the first training phase (September–December 2022), which included workshops on late-life mental health, depression, anxiety, active listening and positive mental health. In January 2023, individual interviews were conducted to select 16 CHWs for the next phase: the iterative pilot. Selection was based on active engagement and lessons from training, prior experience with older adult care, empathy, interpersonal skills, learning aptitude, commitment, and availability. The selected CHWs then participated in the iterative pilot and the subsequent pilot trial (22).

### Data collection

**Figure 2:**
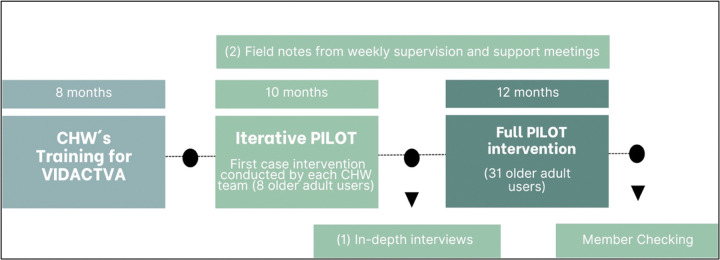
Program implementation stages and data collection timeline including final member checking prodecure

We explored CHWs’ experiences, actions, and perspectives using multiple approaches at different time points (Fig. 2). First, we conducted in-depth interviews with the 16 CHWs after they completed eight sessions with an enrolled older adult. The interview guide was developed for this study by the research team (supplementary file). These interviews, held between September and November 2023, lasted approximately 45 minutes and focused on CHWs’ views regarding the intervention’s acceptability and feasibility, as well as their feedback on program tools and structure. Second, we analyzed field notes from weekly supervision meetings to capture CHWs’ reflections and actions throughout the intervention. These meetings also provided allowed the research team to observe and document the knowledge, resources, and strategies CHWs mobilized in the field that contributed to the program’s overall feasibility.

### Data analysis

We analyzed data using a reflective thematic analysis approach as outlined by (25). An initial codebook was developed inductively by experienced research staff based on the first transcripts, which were transcribed verbatim in Spanish. Codes were then grouped into broader categories and refined into final themes through an iterative process of team discussion and review. In a subsequent step, we examined how the emerging themes related to acceptability, the extent to which a practice, program, or treatment is perceived as agreeable or satisfactory by stakeholders, and feasibility, the extent to which it can be successfully applied within a given context (26, 27). To enhance the trustworthiness and credibility of our findings, we conducted a member-checking procedure with participants to discuss preliminary themes and interpretations (28). Participants were presented with the thematic structure, key quotations, and preliminary analysis. Their feedback was positive, confirming the results and emphasizing two main aspects: the importance of building trustful relationships with participants and the mutual support CHWs provided to one another during the intervention. These insights were integrated into the final analysis presented in this article. Data were managed and organized using MAXQDA software (VERBI GmbH, Berlin, Germany, Version 18.2).

### Ethics

This study received ethical approval from the Institutional Review Boards of *Asociación Benéfica PRISMA* and *Universidad de San Martín de Porres*, both in Lima, Peru. Participants provided written informed consent, granting permission to use interview quotes in academic publications. To ensure confidentiality, all data were de-identified during analysis, with personal identifiers replaced by codes and securely stored in a password-protected, locked online folder. In this manuscript, we use pseudonyms when referring to study participants.

## Results

### Sample characteristics

The study participants consisted of 16 CHWs, ranging in age from 39 to 72 years (Median = 60; SD = 10.4). All female exept for one (CHW 7). They all have lived in VES for over 15 years (Median = 42; SD = 12.6). Most of them had significant years of experience in community leadership, other community-based intervention or programs, and as “*promotoras*” collaborating with the local public health services (Median = 12.5; SD = 11.9). Nevertheless, 11 of them were officially registered as formal CHWs during the VIDACTIVA intervention. For more detailed information about the participants see Table 1.

### Main Themes

We identified three key aspects in the process of piloting and adapting VIDACTIVA that CHWs considered important for strengthening bonds with participants and facilitating engagement and delivery of the program: (1) *establishing trusting relationships*, (2) *adapting session settings and adding tools, and* (3) *supporting one another throughout the intervention.*

### Establish trusting relationships

Active listening and building trustworthy interpersonal connections were essential components of the intervention, especially when working with older adults. CHWs observed that many older adults were hesitant to trust, even with community members like themselves, particularly in neighborhoods affected by crime and violence. They also noted that limited literacy and, at times, deceit by close relatives made older adults vulnerable to scams. One CHW recounted the case of a participant who lost legal ownership of his home after unknowingly signing fraudulent documents.

CHWs collectively recognized that widespread mistrust created significant challenges for implementing VIDACTIVA. They emphasized that earning participants’ trust required sustained effort, sensitivity, and a consistent presence in communities where confidence in both people and institutions had long been undermined. The CHWs described several strategies they used to generate trust with older adults:

#### a. Sharing personal stories and practice “good listening”

CHWs tried to establish trust in different ways. Drawing on their shared experience as long-standing migrants in the area, they often disclosed aspects of their own background and personal history to establish a common ground. They engaged participants in informal conversations about familiar topics such as food and traditions from “*their land*”, as a way to create a sense of connection. These exchanges helped participants to open up about their own personal problems and life challenges, which is seen as an essential step in the VIDACTIVA sessions:

I told her about my town, and she tells me how was hers… (…) I always begin telling my story to engage the person. Then I say “and what has happened to you Mamita?” (…) In that way we were able to know more about the participant, about her problems at home. (CHW 7)

Well, the bond with her developed thanks to the trust she put on us. From the beginning we listened actively to her story, giving her advice and guidance. That helped a lot in her exploration about personal problems (CHW 15)

Actively listening played a critical role in bonding and in further exploration of participants personal problems. “*Build trust. Show them that we are listening and that they can have confidence in us for next time.”*CHWs focused on listening to the participant explaining that “*they [older adults] want to be heard*”. For CHWs, listening was key for establishing trust and rapport with the participants so they could build a solid commitment for the duration of the intervention. They referred that by listening, a participant could change from being avoidant with them to become eager for their scheduled visit:

Listening to him was the way. He had a need for being heard. For example, the first times we knock on his door, he was watching us from the window and not opening, but the last times, he was waiting outside eager to welcome us. (CHW 8)

#### b. Nurturing a family-like approach and friendship

CHWs described showing care and genuine affection by taking a real interest in each person: *“With every user, a hug, a smile, putting interest in the person, their health, what they did last week for example*” (CHW 7). CHWs empahzied that offering love and maintaining regular visits with a reliable presence helped participants to feel valued and supported, much like the care one would expect from a close family member:

We hug her, give her affection, ýou are so pretty, look how much you have accomplished, is so good´. We arrived there as family (…) these persons are in need? of affection, when you give it them, they feel valued, they feel good, they feel like they matter.” (CHW 5)

CHWs also described using an informal form of “laugh therapy” at the beginning of sessions, before discussing more difficult topics. By creating a joyful and trusting environment, they encouraged participants’ self-exploration. CHWs noted that this approach helped participants engage more effectively in the problem-solving exercise:

First, we applied “laugh therapy”. We laughed a lot and then she talked about a traumatic experience from her childhood and right after that she started to think better about pros and cons in the problem-solving exercise (CHW 14)

Among other resources used by CHWs, they mentioned that to develop trust, they presented themselves as friends and not as health workers: *“A friendship. She sends us messages checking in [by phone], sharing something. We respond in a similar way, sharing good desires”* (CHW 12).

#### c. Addressing urgent needs

In their way to develop trust and establish bonds during the intervention, CHWs took concrete actions to meet some urgent needs presented by older adults and considered this to be key for VIDACTIVA intervention: “*Everything is about trust (…) we have to show them that they matter to us*” (CHW 12). They arranged medical appointments, gave orientation about social services, took blood pressure measures, instructed about procedures for bedsores homecare, and implemented local relaxation techniques. Although VIDACTIVA did not include direct assistance in its design, sometimes CHWs chose to provide this support on their own initiative, seeing it as essential to building trust and working effectively with some participants.

During a supervision meeting, CHWs 7 and 12 reported that one participant, who had shown little engagement with VIDACTIVA, was close to dropping out. In response, they decided to address a concrete need the participant had repeatedly voiced: securing a medical appointment at the local health center. The CHWs recalled her frustration, quoting her: *“You don’t give any solution to my problems. My son tells me, ‘Why are they visiting you and asking so many questions? Tell them to help you. What are they giving you?’”* To respond to this concern, CHWs used their knowledge of the health system and personal connections to obtain the needed medical appointment. CHWs believed that this gesture helped restore the participant’s trust in both them and the intervention, ultimately preventing her withdrawal from the program. Other CHWs present at the supervision meeting acknowledged the impact of this out-of-script action, remarking *“They saved the case”* in recognition of the supportive relationship that CHWs 7 and 12 had built through their empathetic and pragmatic response.

In similar circumstances CHWs talked about how they deliver orientation to participants about different topics, such as local and national social services and programs. CHWs refer to this direct assistance orientation as something essential to their community field work: “*If we don’t say anything or orient in any way, how can they trust us? I mean, we always must be focused on serving the community*” (CHW 5). Simlarly a case when the CHWs helped to arrange a medical appointment:

On the first visit we were rejected, but in the second he talked about a needed medication that wasn’t getting from the health center, and we arranged to go there, look for a doctor and bring him the medication so that he can have confidence. So, I think that generating trust in the user is very important. What we do towards the community is generate trust with someone we want to continue to have their friendship or, as in this case to the user, continue the programmed visits (CHW 12)

### Adjust the sessions setting and add tools

Adjusting the VIDACTIVA session’s setting was recognized by CHWs as an important asset to acceptability and feasibility for the program. The CHWs in charge of delivering the intervention freely adapted the basic conditions of the sessions according to the context.

#### a. Session’s location:

Although VIDACTIVA was originally designed as a home-based program, CHWs occasionally shifted sessions from the older adult’s home to nearby public spaces (e.g., a local park) according to participants’ preferences and needs. These adjustments were often motivated by concerns about comfort, privacy, or household conditions such as the presence of family members, the closeness of rooms, or the lack of proper doors, that limited participants’ ability to speak freely and confidentially. By adapting in this way, CHWs followed a participant-centered approach aimed at making sessions more accessible and meaningful:

We told her, ´Miss, should we do the sessions at home?´ And she replied Ńo, because of my family, my sons are there, I don’t want to interrupt. My feelings are for outside. What if we go to the park? ´. ´Which park?´ we replied… So, we did the third session in the park, and then all the rest of them. We said to her, ´the program does not tell us to go out from your home, but it is fine, we will go anywaý. She was so delighted. Till now we write to each other.” (CHW 7)

#### b. New tools for the session:

CHWs resourcefully introduced tools beyond the basic program structure to address challenges that arose during sessions. For instance, CHW 6 proposed a knitting lesson for a reluctant participant with whom conversation was stalling. During supervision, the CHW explained that as they learned a specific stitch together, the dialogue began to flow more naturally, which in turn facilitated the exploration of problems needed to achieve the session’s objectives. In this way, the added activity functioned as a tool to stimulate conversation and led to a favorable outcome.Another tool proposed by the CHWs was a handmade reminder created for a participant who would frequently forget about the scheduled program sessions. Recognizing that the continuity of the intervention was at risk, a CHW tore a piece of paper from their field notebook and wrote, in large letters, the day and time of the next home visit. The participant was encouraged to place the reminder in a visible location within their home to avoid missing future sessions. During a supervision meeting, CHWs later reported that this simple strategy was effective in supporting the participant’s engagement and helped sustain the continuity of care throughout the intervention.

### Support each other throughout the intervention – the supervision “space”

Focusing on CHWs’ dynamics, we found that the development of a supportive peer group was a key factor contributing to the intervention’s acceptability. What began as weekly supervision meetings with a clinical and community psychologist and a general practitioner gradually evolved into much more than a technical space. Over nearly two years (2022–2024), the 16 CHWs shared their field experiences, reflected on challenges, and supported one another. Through this process, they forged a strong sense of team, recognizing the supervision space as not only a forum for professional guidance but also a form of self-care that fostered solidarity, mutual support, and trust among them.

CHWs often found themselves needing information, advice, or other inputs to better support their participants. In this context, CHW 6 highlighted the value of peer support and the collective approach fostered by the intervention, recognizing it as an important part of her training and overall experience:

First, they (research team) met us, they explained the program to us, they trained us, (...) then they organized us in couples and then we came and held our meetings explaining, telling how it had gone, our experiences, and that enriched our learning a lot (CHW 6)

CHWs highlighted that belonging to the group was especially helpful, as peer inputs during supervision played a crucial role in building their confidence and skills. While guidance from the research team was valuable, the support and shared knowledge among peers often proved even more important for their ongoing learning and implementation:

At the beginning, you know that we have not done things right. But along the way, with the contribution of the our colleagues and of you really, one has begun to improve, to get on the right track. Also, since we have had several users, we already have a little more experience, more confidence. We have built that with the user as well as we have gained it for ourselves, and we already feel safer. (…) That is why the supervision sessions that we have every Thursday are very valuable, very important, for our learning. (CHW 10)

We found that hearing about colleagues’ experiences and challenges strengthened CHWs for their fieldwork. The confidence gained from these weekly meetings contributed to recognizing themselves as a learning group, consolidating their sense of unity and collective support:

Of course, the fact that after sharing here, listening to the other colleagues going through the same thing, (...) the fact of sharing here helps one a lot, doesn’t it? (...) listening to others also strengthens us, it continues to strengthen us, and when we start with the new user, we are now calmer, more convinced. (CHW 11)

This is sharing. Since we have started, after each session we get together and each one of us can share their experience. (…) So, we feel together, united and sharing in every meeting, gaining a lesson and leaving a lesson. (CHW 12)

## Discussion

Our study describes the actions, experiences, insights, and resources mobilized by CHWs during the intervention, which helped strengthen bonds with older adults. First, CHWs emphasized the importance of establishing trusting relationships, which they nurtured through active listening, sharing personal stories, showing affection, and, at times, addressing urgent needs. Second, they adapted the program by adjusting session settings and incorporating creative activities to overcome barriers to engagement and sustain participation. Finally, weekly supervision sessions evolved into spaces of peer learning, solidarity, and self-care that strengthened CHWs’ confidence and sense of teamwork.

Building trusting relationships emerged as central to CHWs’ performance in the field, making sessions more viable, accepted, and appreciated by participants. Previous studies have similarly emphasized that trust is fundamental for effective CHW engagement (29, 30). Trust toward health providers has also been identified as a critical factor when implementing community-based interventions, particularly in contexts affected by violence (15, 31, 32). Rodríguez Gómez (33) further notes that bonds based on affection and trust are pillars for consolidating social relationships within communities. In addition, Sullivan et al. (34) highlight that support and care among CHWs contribute to their emotional and mental well-being, positively influencing their self-efficacy, relationships with peers, and interactions with patients.

Community-based programs such as VIDACTIVA benefit from allowing adaptations that draw on CHWs’ knowledge and experience within their communities. Granting CHWs the flexibility to adjust sessions and build trust in their own ways proved decisive for adapting the intervention. Evidence suggests that when supervised and applied appropriately, peer self-disclosure can enhance depression care for older adults by fostering rapport, empathy, and understanding of personal challenges (35). Although this manuscript does not report older adults’ perspectives, evidence from other contexts shows that participants valued CHWs’ peer self-disclosure, cultural closeness, and practical support (36). Such elements are less common in interactions with regular health providers, who are generally trained to avoid personal disclosure in order to maintain professional boundaries.

We consider the close and sustained relationship built with the CHW team during weekly meetings over three years to be a major strength of this study. These ongoing interactions not only fostered strong bonds between CHWs and supervisors but also provided a unique opportunity to observe the team’s dynamics over time. This long-term immersion, centered on the adaptation and implementation of VIDACTIVA, enabled an in-depth understanding of CHWs’ knowledge, resources, and limitations, an approach that reflects the value of prolonged engagement in qualitative research for capturing the complexity of a phenomenon (37).

As with any study, there are limitations to consider. In-depth interviews may have been perceived by CHWs as a form of evaluation following the development phase of VIDACTIVA, which could have influenced their responses through social desirability bias. Similarly, during supervision sessions, social desirability may have shaped how CHWs described their interactions with participants and with one another. Member-checking also carried a potential risk, as CHWs may have been reluctant to contradict the researcher leading the project. Nevertheless, the openness with which CHWs shared difficult or negative situations with older adults suggests that the impact of these biases was likely limited.

## Conclusions

After examining CHWs’ actions, experiences, knowledge, and resources, we emphasize that in community-based interventions and research, establishing trusting relationships is a critical strategy for fostering participant engagement and facilitating delivery, something CHWs in this study consistently demonstrated in practice. Building and sustaining trust within the community and with individuals should therefore be a core consideration in intervention design, guiding both the development and implementation of community-based programs.

We highlight the importance of recognizing and leveraging CHWs’ experience and knowledge as a critical factor in conducting community-based interventions. Doing so makes interventions more humanized, organic, and emotionally closer to participants, while at the same time promoting the natural use of personal and social resources already present within the community.

Finally, community-based interventions that work with CHWs should not only view them as individual workers or supervised pairs, but also as a collective with the potential to cultivate relationships among themselves. Creating spaces where CHWs can share experiences, support one another, and engage in self-care strengthens their confidence and sense of teamwork. This collective dimension is not only key to learning and mutual support but may also provide a foundation for the long-term sustainability of community-based programs.

## Supplementary Material

Supplementary Files

This is a list of supplementary fi les associated with this preprint. Click to download.

• InterviewguideforCHWsPostFase2.pdf

## Figures and Tables

**Table 1:** CHW’s sociodemographic, education and community service information

	Age in years	Born in Lima (Yes/No)	Marital status	Living with whom	Years living in *Villa el Salvador*	Level of Education	Experience as community leader in years
CHW 1	66	No	Married	Husband	25	Technical qualification	25
CHW 2	38	Yes	Single	Children	20	Technical qualification	30
CHW 3	59	Yes	Married	Husband and children	52	Completed High school	7
CHW 4	42	Yes	Single	Mother and children	37	Technical qualification	7
CHW 5	65	No	Married	Husband, children and son in law	51	Completed High school	40
CHW 6	63	Yes	Divorced	Children and grandchildren	36	Technical qualification	10
CHW 7	74	No	NA	Partner and niece	50	Completed High school	20
CHW 8	52	No	Life Partner	Partner, children and grandchildren	30	completed High school	10
CHW 9	50	No	Married	Husband and children	22	completed High school	8
CHW 10	75	Yes	Widow	Children, sons and daughters in law and grandchildren	46	Bachelor’s degree	20
CHW 11	67	Yes	Widow	Children and godson	49	Bachelor’s degree	24
CHW 12	63	Yes	Single	Sister, children and grandchildren	50	Technical qualification	40
CHW 13	61	No	Married	Husband, children and grandchildren	38	Completed High school	7
CHW 14	57	Yes	Divorced	Parents	49	Technical qualification	15
CHW 15	49	Yes	Married	Husband and children	16	Bachelor’s degree	3
CHW 16	56	Yes	Divorced	Children	50	Completed High school	8

## Data Availability

The datasets used and/or analysed during the current study are available from the corresponding author on reasonable request.

## Abbreviations

CHW: Community Health Worker
PEARLS: Program to Encourage Active, Rewarding Lives
VES: Villa El Salvador
PHC: Primary Healthcare Center
CMHC: community mental health centers
